# Trends in Outpatient Care and Use of Telemedicine After Hospital Discharge in a Large Commercially Insured Population

**DOI:** 10.1001/jamahealthforum.2021.3685

**Published:** 2021-11-12

**Authors:** Eric Bressman, Ali Russo, Rachel M. Werner

**Affiliations:** 1 Department of Medicine, Perelman School of Medicine, University of Pennsylvania, Philadelphia; 2 Leonard Davis Institute of Health Economics, University of Pennsylvania, Philadelphia; 3 Corporal Michael J. Crescenz VA Medical Center, Philadelphia, Pennsylvania; 4 FAIR Health, Inc, New York, New York

## Abstract

This cross-sectional study examines changes in use of telemedicine vs in-person care among a commercially insured population durring the COVID-19 pandemic.

## Introduction

The period after hospital discharge is a vulnerable time for patients, and timely access to outpatient care is an important component of safe transitions to the home setting, particularly for older adults.^1^ The expansion of telemedicine in response to the COVID-19 pandemic opened a new avenue for accessing care. It has also raised questions around the cost and quality of telemedicine services, particularly as policy makers consider extending coverage mandates for telehealth.^2^ Two important, related questions are whether telemedicine increases access to care and whether it substitutes for or adds to in-person care. The rapid growth of telemedicine provides the opportunity for new insights into these questions and the opportunity to inform payment decisions moving forward. To address these questions, we looked at trends in utilization of outpatient care after hospital discharge.

## Methods

We used multipayer, deidentified, noncapitated claims data from FAIR Health from January 2019 to December 2020. These longitudinal data include over 70 million commercially insured and Medicare Advantage enrollees. We identified all hospital discharges to home for patients aged 50 years and older, and then identified all evaluation and management (EM) visits (both in-person and telemedicine) with a primary care, medical subspecialty, or surgical specialty clinician during the 30 days after discharge. We measured care access by calculating the monthly percent of discharges with 1 or more EM visits within 30 days. We measured utilization of care by calculating the mean number of visits per discharge within 30 days.

This study was reviewed by the University of Pennsylvania institutional review board and categorized as exempt because all data used were deidentified. Analyses were conducted using Oracle Advanced Analytics in the Oracle Database 19c. The analysis took place from April 12, 2021, to June 11, 2021. More details are available in the eMethods in the Supplement.

## Results

**Figure 1.**
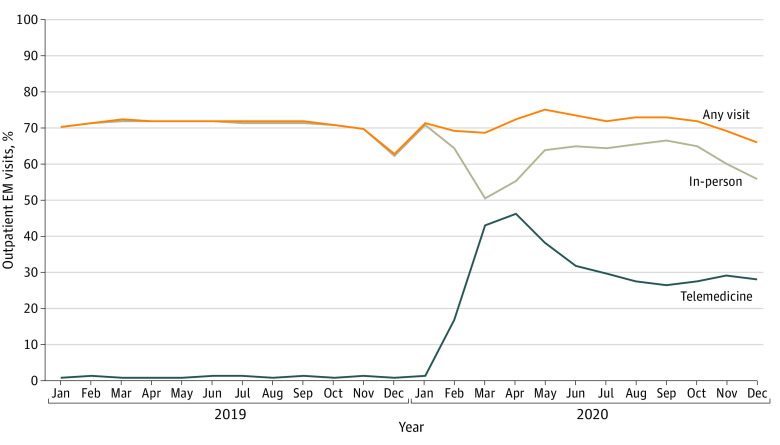
Trends in Discharged Enrollees Completing an Outpatient Evaluation and Management (EM) Visit During the 30 Days Postdischarge The trend lines represent the percent of discharged enrollees that completed 1 or more outpatient EM visits within 30 days of discharge, including in-person (gray), telemedicine (blue), or any type of visit (orange).

We analyzed 1 616 566 hospital discharges. The percent of discharges with an in-person visit dropped from 72% in April 2019 to 55% in April 2020, whereas those with a telemedicine visit increased from 0% to 46% over the same period (Figure 1). The percent of discharges with any type of visit increased from an average of 72% before the pandemic to a high of 75% in May 2020 but declined again to levels similar to before the pandemic.

**Figure 2.**
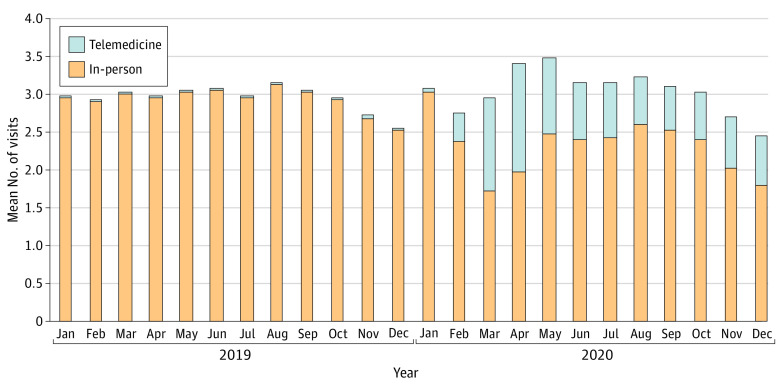
Trends in Utilization of Outpatient Care Postdischarge The stacked bars represent mean total outpatient evaluation and management visits within 30 days of discharge per discharged enrollee, including in-person visits (orange) and telemedicine visits (gray).

The mean number of EM visits per discharge within 30 days increased from 2.98 in April 2019 to 3.40 in April 2020 but then regressed toward baseline levels (Figure 2). The mean number of in-person visits went from 2.94 in 2019 to 2.35 in 2020, a decrease of 0.6. Telemedicine visits made up for this difference, increasing from 0.02 in 2019 to 0.70 in 2020, an increase of 0.7.

## Discussion

In this large, commercially insured population, overall use of outpatient visits after hospital discharge did not change significantly, even as telemedicine use increased. The percentage of patients completing a postdischarge visit stayed around 70%—consistent with findings prior to the pandemic^3^—even as general ambulatory visits dropped significantly.

Notably, although the mean number of visits after discharge did not change, telemedicine appeared to substitute for in-person visits. Prepandemic research suggested telemedicine was more likely additive to in-person care for certain clinical conditions.^4^ Data on outpatient visits early in the pandemic showed considerable declines in volume as discretionary care was deferred, with telemedicine compensating for some of this drop.^5^ Telemedicine offers added convenience for patients recovering from a hospitalization and postdischarge follow-up care is less likely to be considered discretionary, making it a useful case study in utilization trends.

As state and federal emergency waivers expire, policy makers will need to decide which telemedicine services deserve ongoing coverage, and at what level.^6^ Although there has been concern that payment parity may lead to overutilization, our research suggests that overall postdischarge utilization has not been significantly affected, whereas nearly 30% of those discharged have continued to use telemedicine. This work may not be generalizable beyond the commercially insured population studied. We attempted to mitigate the effect of pandemic-related disruptions to usual care by focusing on nondiscretionary visits. Although care access, broadly defined, has not been affected, future work should look at whether the sociodemographic distribution of who is accessing care has changed.

## Notes

Supplement.
eMethods
