# Supplementary material for: Trends in Outpatient Care and Use of Telemedicine After Hospital Discharge in a Large Commercially Insured Population
Source: JAMA Health Forum. 2021 Nov 12;2(11):e213685. doi: 10.1001/jamahealthforum.2021.3685 (PMC8796902; doi:10.1001/jamahealthforum.2021.3685)
Supplement: Supplement. — eMethods [file jamahealthforum-e213685-s001.pdf]

Supplemental Online Content

Bressman E, Russo A, Werner RM. Trends in outpatient care and use of telemedicine after hospital discharge in a large commercially insured population. *JAMA Health Forum*. 2021;2(11). doi:10.1001/jamahealthforum.2021.3685

eMethods.

This supplemental material has been provided by the authors to give readers additional information about their work.

© 2021 Bressman E, et al. *JAMA Health Forum.*

Telehealth services were identified as those services which were billed using any of the following criteria:

- Place of service of 02
- Procedure code modifiers of 95, G0, GQ or GT
- Telehealth procedure codes:

☐

Procedure Code	Description	Procedure Code	Description
98966	NON-FACE-TO-FACE NONPHYSICIAN TELEPHONE SERVICES	99473	REMOTE PHYS MONITORING
98967	NON-FACE-TO-FACE NONPHYSICIAN TELEPHONE SERVICES	99474	REMOTE PHYS MONITORING
98968	NON-FACE-TO-FACE NONPHYSICIAN TELEPHONE SERVICES	G0181	HOME HEALTH CARE SUPERVISION
98969	ONLINE DIGITAL E&M SERVICES	G0182	HOSPICE CARE SUPERVISION
98970	NON-FACE-TO-FACE NONPHYSICIAN ONLINE E&M SERVICES	G0406	INPATIENT CONSULTATION - TELEHEALTH
98971	NON-FACE-TO-FACE NONPHYSICIAN ONLINE E&M SERVICES	G0407	INPATIENT CONSULTATION - TELEHEALTH
98972	NON-FACE-TO-FACE NONPHYSICIAN ONLINE E&M SERVICES	G0408	INPATIENT CONSULTATION - TELEHEALTH
99091	REMOTE PHYS MONITORING	G0425	EMERGENCY ROOM/INPATIENT - TELEHEALTH
99421	ONLINE DIGITAL E&M SERVICES	G0426	EMERGENCY ROOM/INPATIENT - TELEHEALTH
99422	ONLINE DIGITAL E&M SERVICES	G0427	EMERGENCY ROOM/INPATIENT - TELEHEALTH
99423	ONLINE DIGITAL E&M SERVICES	G0459	INPATIENT PHARM MANAGEMENT - TELEHEALTH
99441	NON-FACE-TO-FACE TELEPHONE SERVICES	G0508	CRITICAL CARE - TELEHEALTH
99442	NON-FACE-TO-FACE TELEPHONE SERVICES	G0509	CRITICAL CARE - TELEHEALTH
99443	NON-FACE-TO-FACE TELEPHONE SERVICES	G2010	REMOTE IMAGE/VIDEO EVALUATION
99444	ONLINE DIGITAL E&M SERVICES	G2012	VIRTUAL CHECK IN BY PHYS OR QUAL HEALTH CARE PROF E&M
99453	REMOTE PHYS MONITORING	G2061	QUAL NONPHYS HEALTH PROF ONLINE ASSESS & MANAGEMENT SVC, EST PT 5-10M
99454	REMOTE PHYS MONITORING	G2062	QUAL NONPHYS HEALTH PROF ONLINE ASSESS & MANAGEMENT SVC, EST PT 11-20M
99457	REMOTE PHYS MONITORING	G2063	QUAL NONPHYS HEALTH PROF ONLINE ASSESS & MANAGEMENT SVC, EST PT >21M
99458	REMOTE PHYS MONITORING		

© 2021 Bressman E, et al. *JAMA Health Forum.*
